# Evaluation of Hepatoprotective Effect of Leaves of *Cassia sophera* Linn.

**DOI:** 10.1155/2012/436139

**Published:** 2012-05-29

**Authors:** Arijit Mondal, Sanjay Kumar Karan, Tanushree Singha, D. Rajalingam, Tapan Kumar Maity

**Affiliations:** ^1^Department of Pharmaceutical Technology, Jadavpur University, Jadavpur, West Bengal, Kolkata 700 032, India; ^2^Seemanta Institute of Pharmaceutical Sciences, Jharpokharia, Odhisa 757 086, India

## Abstract

In the present study, the hepatoprotective activity of ethanolic extracts of *Cassia sophera* Linn. leaves was evaluated against carbon-tetrachloride- (CCl_4_-) induced hepatic damage in rats. The extracts at doses of 200 and 400 mg/kg were administered orally once daily. The hepatoprotection was assessed in terms of reduction in histological damage, changes in serum enzymes, serum glutamate oxaloacetate transaminase (AST), serum glutamate pyruvate transaminase (ALT), serum alkaline phosphatase (ALP), total bilirubin, and total protein levels. The substantially elevated serum enzymatic levels of AST, ALT, ALP, and total bilirubin were restored towards the normalization significantly by the extracts. The decreased serum total protein level was significantly normalized. Silymarin was used as standard reference and exhibited significant hepatoprotective activity against carbon tetrachloride-induced hepatotoxicity in rats. The biochemical observations were supplemented with histopathological examination of rat liver sections. The results of this study strongly indicate that *Cassia sophera* leaves have potent hepatoprotective action against carbon tetrachloride-induced hepatic damage in rats. This study suggests that possible activity may be due to the presence of flavonoids in the extracts.

## 1. Introduction

*Cassia sophera* Linn (Family Caesalpiniaceae), popularly known as kasundi, is a shrubby herb found throughout India and in most tropical countries. In the ethnobotanical claims, the leaves are considered to be used for their anti-inflammatory, antirheumatic, and purgative property, as an expectorant for cough, cold, bronchitis, and asthma, and in the treatment of liver disorders. Previous studies have investigated on its pharmacological activities of the seeds of *C. sophera* including analgesic and anticonvulsant [1], antidiabetic [2], inhibition of lipid peroxidation [3], herbicidal [4], and fungicidal [5] effects.

The chemical constituents of *C. sophera* include the flavonoids [6, 7] and anthraquinone [8, 9]. To the best of our knowledge, there is no scientific report of hepatoprotective effect of *C. sophera*. Thus, the present study was to investigate the hepatoprotective activity of ethanol extract of leaves of *C. sophera* against CCl_4_-induced hepatic damage in rats.

## 2. Materials and Methods

### 2.1. Plant Material

The fresh leaves of *Cassia sophera* Linn was collected from Tiruvannamalai district of Tamilnadu, India, in October and November. The plant was identified by B. Velmurugan, Taxonomist, Sri Ramana Maharishi Natural Society, Tiruvannamalai, India. A voucher specimen (Reg. no. GPT/8/2003) was deposited in our laboratory for future references. The leaves of the plant were dried under the shade and then milled into coarse powder, stored in an air tight closed container.

### 2.2. Extraction and Isolation

The dried coarse powdered *Cassia sophera* leaves (1.5 kg) were first defatted with petroleum ether (60–80°C) and then extracted with 5 L of ethanol (90%) in a soxhlet apparatus. The solvent was then removed under reduced pressure, to obtain petroleum ether (PECS, yield 8.5%) and ethanol extract (EECS, yield 22.5%), respectively. The ethanol extract was partitioned successively between chloroform and ethyl acetate (3 × 1 L). The respective solvents were removed similarly under reduced pressure, which produced ethyl acetate fraction (EAF) (150 g) and chloroform fraction (CF) (50 g). Both fractions were evaluated for hepatoprotective activity against CCl_4_-induced hepatic damage in rats. EAF was found to be more potent than CF. Hence, EAF was further exploited for isolation, which led to the isolation of rhamnetin, O-methylated flavonol. The isolated bioactive metabolite was characterized as rhamnetin based on melting point and spectroscopic (IR, ^1^H NMR and MS) data [10, 11].

 7 g of the ethyl acetate fraction was adsorbed on silica gel (silica gel 60 G, Merck, 600 g) and applied to a column of silica gel. A gradient of chloroform : ethyl acetate : methanol was used to elute the column, collecting 100 fractions of 50 mL each. Fractions, 35–42, were combined and, on TLC, it shows a single spot having an *R*_*f*_ value of 0.58. These combined fractions are evaporated to dryness and were further rechromatographed on a silica gel column using a gradient elution with chloroform : ethyl acetate (8 : 2) to give one compound, which was recrystallized with methanol to give pure rhamnetin.

### 2.3. Animals

Adult male Wistar albino rats weighing 150–180 g were used for the present investigation. All animal experiments were duly approved by Institutional Ethical Committee (CPCSEA/ORG/CH/2006/Reg. no.95), Jadavpur University, Kolkata, India.

### 2.4. Chemicals and Drugs

Silymarin was purchased from Microlabs (Hosur, Tamilnadu, India), carbon tetrachloride purchased from SICCO Research Laboratory, Mumbai, India. All other chemicals and solvent were of analytical grade and commercially available.

### 2.5. Acute Toxicity Test

The animals were divided into five groups (*n* = 6). The EECS suspension was administrated orally in increasing dose up to 2000 mg/kg, b.w [12]. The rats were observed continuously for 2 h for behavioural, neurological, and autonomic profiles and after 24 and 72 h for any lethality [13].

### 2.6. Experimental Design

The animals were divided into five groups (*n* = 6). Group I served as a vehicle control, which received liquid paraffin, intraperitoneally. Groups II–V were treated with CCl_4_ in liquid paraffin (1 : 2) at the dose of 1 mL/kg body weight (b.w) intraperitoneally once in every 72 h for 16 days [14]. Aqueous suspension of EECS at the doses of 200 mg/kg and 400 mg/kg, b.w, were administered orally to the animals in groups III to IV in alternate days for 16 days. Group V received silymarin as a standard drug at the dose of 25 mg/kg, b.w., p.o. in alternate days for 16 days. At the 17th day, all the rats were sacrificed by cervical dislocation after collecting the blood from retroorbital plexus under ether anesthesia for biochemical estimations. The blood samples were allowed to clot and the serum was separated by centrifugation at 5000 rpm for 5 min and used for the assay of biochemical marker enzymes.

### 2.7. Biochemical Estimations

Different biochemical parameters like aspartate transaminase (AST), alanine transaminase (ALT), alkaline phosphatase (ALP), total bilirubin and total protein were determined by using commercially available kits (Span Diagnostic Limited, Surat, India).

### 2.8. Histological Observation

The washed liver tissues were fixed by using fixative (picric acid, formaldehyde, and 40% glacial acetic acid) for 24 h and dehydrated with alcohol. Liver tissues were cleaned and embedded in paraffin (melting point 58–60°C), cut in 3–5 *μ*m sections, stained with the haematoxylin-eosin dye and finally, observed under a photomicroscope and morphological changes such as cell necrosis, ballooning degeneration, fatty changes or inflammation of lymphocytes were observed [15].

### 2.9. Statistical Analysis

The results were analyzed from statistical significance by one-way analysis of variance (ANOVA) followed by Dunnett's post hoc test using Statistical Package of the Social Science (SPSS) software. Results are expressed as mean ± SD for six rats in each group. Differences among groups were considered significant at *P* < 0.05 level.

## 3. Results

### 3.1. Phytochemical Screening and Isolation of Rhamnetin

**Figure 1:**
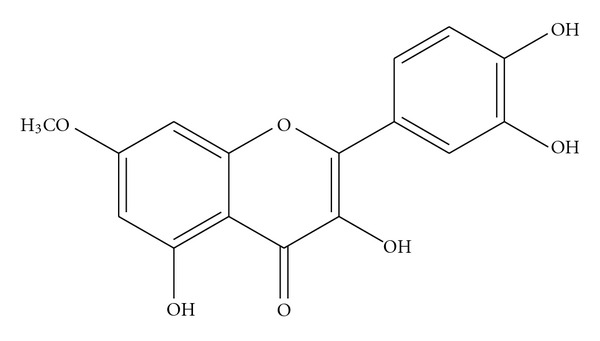
Structure of Rhamnetin.

Preliminary phytochemical screening of the ethanol extract of *C. sophera* revealed the presence of steroids, alkaloids, tannins, saponins, and flavonoids. Different compositions of the mobile phase were tested and the desired resolution of rhamnetin with symmetrical and reproducible peak was achieved by using the mobile phase chloroform and ethyl acetate. The structure of the compound was characterized by UV, IR, MS, and 13C-NMR methods as rhamnetin (Figure 1). Structures and the IR, 13C-NMR, and MS data obtained independently in these studies are in close conformity with reported literature [10, 16].

yellow colour crystals, TLC: (chloroform : methanol, 9 : 1 v/v) *R*_*f*_ 0.59: UV *λ*_max⁡_ (C_2_H_5_OH): 360.1 nm; m.p. 282–285°C. MS m/z 316 (calculated value C_16_H_12_O_7_, 316.26). ^1^H NMR (CD_3_OD): *δ* 13.04 (s, 1H, OH-5), *δ* 12.96 (s, 2H, OH-3′, 4′), *δ* 7.32–7.41 (d, 1H, H-6′), *δ* 6.88 (d, 1H, *J* = 1.2, H-5′), *δ* 6.50 (d, 1H, *J* = 2, H-8), *δ* 6.43 (d, 1H, *J* = 2, H-6), *δ* 3.78 (s, 3H, OCH_3_). The proton signal at *δ* 3.78 (s, 3H, OCH_3_) suggests the location of –OCH_3_ at C-7, IR (KBr) v cm^−1^, 3388 (O–H), 1654 (>C=O), 1610 (C=O), 1029 (C–O–C).

### 3.2. Acute Toxicity Studies

Acute toxicity studies revealed the nontoxic nature of the ethanol extracts of *Cassia sophera*. There was no lethality or toxic reaction found at any doses selected until the end of the study period.

### 3.3. Hepatoprotective Activity

Rats treated with CCl_4_ developed a significant hepatic damage and oxidative stress. This is evident to the significant (*P* < 0.05) increase in serum ALT, AST, ALP, and bilirubin levels in CCl_4_-treated rats compared to normal rats. However, the serum total protein level was significantly (*P* < 0.05) decreased in CCl_4_-intoxicated rats. The toxic effects of CCl_4_ were controlled in the animals treated with methanol extract of *Cassia sophera* at the doses of 200 and 400 mg/kg, p.o. significantly (*P* < 0.05) decreased the elevated serum marker enzymes. Total bilirubin and total proteins were found to be restored to almost normal level. The effects of EECS on serum ALT, AST, ALP, bilirubin and total protein levels in CCl_4_ intoxicated rats are summarized in Table 1.

**Figure 2:**
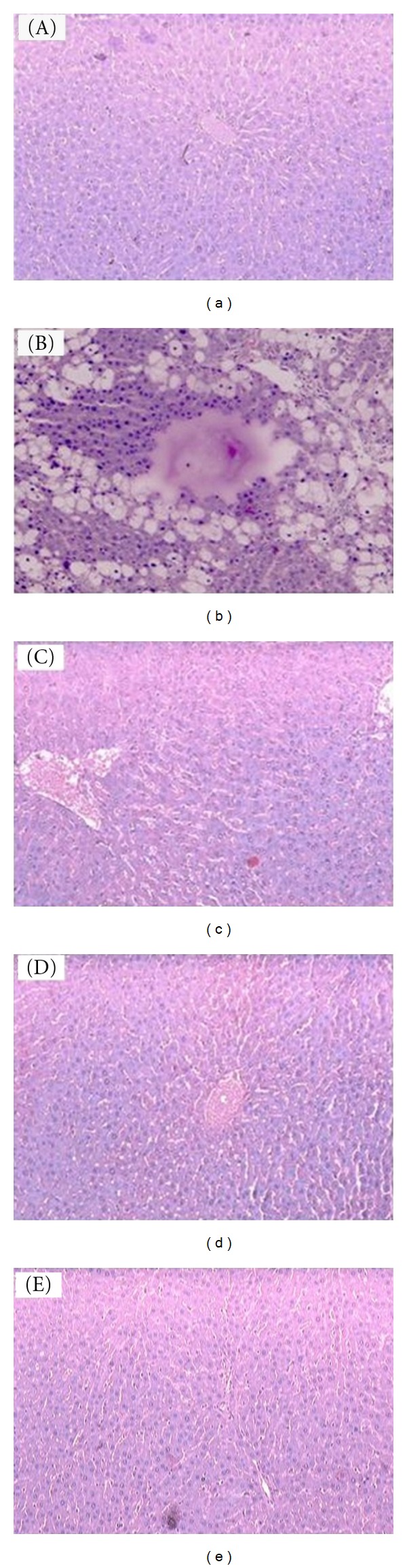
Photomicroscopy of liver sections from CCl_4_-intoxicated rats (10x). Histological observations of the liver tissue of the normal animals showed hepatic cells with well-preserved cytoplasm, nucleus, nucleolus, and central vein (a). Treatment with CCl_4_ caused fatty degeneration with severe necrosis of the parenchyma cells in the central lobular region of the liver. Furthermore, hepatocytic necrosis was predominant surrounding the central vein, which formed a streak-like appearance (b), (c), (d), and (e) showed animals treated with EECS (200 and 400 mg/kg, p.o.) and silymarin (25 mg/kg) and restored the altered histopathological changes, respectively.

Histological observations of the liver tissue of the normal animals showed hepatic cells with well-preserved cytoplasm, nucleus, nucleolus, and central vein (Figure 2(a); 10x). Treatment with CCl_4_ caused fatty degeneration with severe necrosis of the parenchyma cells in the central lobular region of the liver. Furthermore, hepatocytic necrosis was predominant surrounding the central vein, which formed a streak-like appearance (Figure 2(b); 10x). Figures 2(c), 2(d) and 2(e) (10x) show animals treated with EECS (200 and 400 mg/kg, p.o.) and Silymarin (25 mg/kg) and restored the altered histopathological changes, respectively.

## 4. Discussion

Preventive action in liver damage induced by carbon tetrachloride has widely been used as an indicator of the liver protective activity of drugs in general [17]. It was found that chronic administration of CCl_4_ produces liver cirrhosis in rats. It is well documented that carbon tetrachloride is biotransformed under the action of cytochrome P-450 system in the microsomal compartment of liver to trichloromethyl or peroxytrichloromethyl free radical. These free radicals bind covalently to the macromolecules and induce peroxidative degradation of the membrane lipids of endoplasmic reticulum rich in polyunsaturated fatty acids. This leads to the formation of lipid peroxides followed by pathological changes such as triacylglycerol accumulation, polyribosomal disaggregation, depression of protein synthesis, cell membrane breakdown, and even death [18, 19].

Estimating the activities of serum marker enzymes like AST, ALT, ALP, and bilirubin can make assessment of liver function. When liver cell plasma membrane is damaged, a variety of enzymes normally located in the cytosol are released into the blood stream. Their estimation in the serum is a useful quantitative marker of the extent and type of hepatocellular damage [20].

 The increased level of AST, ALT, ALP, and bilirubin is conventional indicator of the liver injury. In the present study, it is observed that administration of CCl_4_ elevates the levels of serum marker enzymes AST, ALT, ALP, and bilirubin. Levels of total proteins are lowered. Ethanol extracts of *Cassia sophera* and reference drug silymarin-treated groups exhibited lower levels of AST, ALT, ALP, and bilirubin as compared to CCl_4_ treated groups. The treatment with MECS also significantly elevated total protein levels. The stabilization of serum AST, ALT, ALP and bilirubin by EECS is clear indication of the improvement of the functional status of the liver cells. The characteristic feature of experimental hepatic damage observed is significant decrease in protein level. The rats in group IV, which receive EECS, showed restoration of protein levels.

 These findings can be further corroborated with histopathological studies. The histopathological examination clearly reveals that the hepatic cells, central vein, and portail triad are almost normal in EECS (400 mg/kg. p.o.) group in contrast to group IV, which receive CCl_4_ only. Thus EECS can be considered to be an effective hepatoprotective as it ameliorates almost to normalcy the damage caused by CCl_4_ to hepatic function.

As the flavonoid compound isolated from *Artemisia scorparia* [21] is reported to possess hepatoprotective activity, it is very likely that the flavonoid glycoside in *C. sophera* [22] may be responsible for hepatoprotective activity, but further exploration is needed.

## 5. Conclusion

The ethanolic extract of *Cassia sophera* could effectively control the AST, ALT, ALP, and total bilirubin levels and increase the protein levels in the protective studies. The histopathological studies also substantiate the activity of the drug. Therefore, the study scientifically supports the usage of this plant in traditional medicine for treatment of liver disorders and as a tonic.

## Figures and Tables

**Table 1:** Effect of EECS and silymarin on serum biochemical parameters.

Biochemical parameters	Control	CCl_4_ 1 mL/kg	MECS (200 mg/kg) + CCl_4_	MECS (400 mg/kg) + CCl_4_	Silymarin (50 mg/kg) + CCl_4_
ALT (IU/L)	84 ± 5.4	178.3 ± 10.1^a^	151.6 ± 11.1*	135.3 ± 3.4*	120.0 ± 3.9*
AST (IU/L)	143.3 ± 14.8	251.6 ± 11.9^a^	220.0 ± 11.5*	180.8 ± 13.1*	158.3 ± 10.6*
ALP (IU/L)	82.2 ± 7.8	190.0 ± 13.9^a^	173.3 ± 13.1*	130.5 ± 9.5*	108 ± 5.2*
Total protein (mg/dL)	6.9 ± 0.32	3.5 ± 0.46^a^	4.6 ± 0.28	5.2 ± 0.21**	6.0 ± 0.31
Total bilirubin (mg/dL)	0.29 ± 0.04	0.98 ± 0.07^a^	0.79 ± 0.07	0.63 ± 0.05**	0.48 ± 0.07*

Values are mean ±  SEM (*n* = 6). *P < 0.01 (moderately significant), **P < 0.05 (significant) as compared with CCl_4_, ^a^: significant as compared with control (*P* < 0.01).
